# Supplementary material for: Modeling spatial determinants of initiation of breastfeeding in Ethiopia: A geographically weighted regression analysis
Source: PLoS One. 2022 Sep 15;17(9):e0273793. doi: 10.1371/journal.pone.0273793 (PMC9477376; doi:10.1371/journal.pone.0273793)
Supplement: Attachment — Submitted filename: Point by point respons.docx [file pone.0273793.s002.docx]

**To: PLOS ONE**

From: Samuel Hailegebreal

Subject: **A letter Accompanying Revision in Response to Editors and Reviewer Comments**

**Dear Editors**

The authors would like to thank the editorial team and team of reviewers for constructive and valuable comments. The authors are very happy to submit the second revised version of the manuscript entitled **“Modeling spatial determinants of initiation of breastfeeding in Ethiopia: A geographically weighted regression analysis”** for its publication in your Journal. The comments of the editors and the reviewers were highly insightful and enabled us to greatly improve the quality of our manuscript. In this second revision manuscript we made substantial changes to address your concerns in a point-by-point response. We are very keen to incorporate further comments, if any, for the betterment of the final manuscript.

**Point by Point Response to - Editor Comments**

**Point by Point Response to Reviewers**

Please review your reference list to ensure that it is complete and correct. If you have cited papers that have been retracted, please include the rationale for doing so in the manuscript text, or remove these references and replace them with relevant current references. Any changes to the reference list should be mentioned in the rebuttal letter that accompanies your revised manuscript. If you need to cite a retracted article, indicate the article’s retracted status in the References list and also include a citation and full reference for the retraction notice.

Author response: thank you for your comment. We author updated reverence no 1, 2, 3, 27, 28 in the revised manuscript

**Reviewer #1**

1. It is not clear if the authors used the “fill missing value” tool to complete the dataset with estimated values or if they just excluded the observations with missing value in any of the study variables. In the section “Data management and Statistical analysis”, please clarify how you handled missing data.

**Authors’ response:** Thank you for your input. A variable with a "missing value" should have a response but does not because the question was not asked (due to interviewer error) or the responder did not want to answer. We use the STATA drop command in conjunction with a logical / conditional expression to remove missing values from our analysis.

2. Do you really need all those statistics in table 2 in order to interpret the results? Please leave in the table only the essential statistics.

**Authors’ response:** Thank you for the comment we accept and corrected in the revised version

| **Variable** | **Coefficient** | | **Robust standard error** | | **Robust t statistics** | **Robust probability** | | **VIF** |
| --- | --- | --- | --- | --- | --- | --- | --- | --- |
| Intercept | 0.06 | | 0.020 | | 3.09 | < 0.01 | | ------ |
| Orthodox | 0.12 | | 0.024 | | 5.05 | < 0.01 | | 1.13 |
| Proportion poor | 0.17 | | 0.028 | | 6.09 | < 0.01 | | 1.27 |
| Proportion of cesarean delivery | 0.22 | | 0.079 | | 2.83 | < 0.01 | | 1.12 |
| Proportion of baby postnatal checkup | 0.13 | | 0.063 | | 2.08 | < 0.05 | | 1.09 |
| Proportion of small size at birth | 0.20 | | 0.046 | | 4.24 | < 0.01 | | 1.08 |
| **Ordinary least square regression diagnostics** | | | | | | | | |
| Number of Observations: | | 611 | | Adjusted R-Squared | | | 0.137 | |
| Joint F-Statistic | | 20.37 | | Prob(>F), (5,605) degrees of freedom | | | < 0.01 | |
| Joint Wald Statistic: | | 100.27 | | Prob(>chi-squared), (5) degrees of freedom | | | < 0.01 | |
| Koenker (BP) Statistic | | 22.100 | | Prob(>chi-squared), (5) degrees of freedom | | | < 0.01 | |
| Jarque-Bera Statistic | | 39.838 | | Prob(>chi-squared), (2) degrees of freedom | | | < 0.01 | |
