# Supplementary material for: Modeling spatial determinants of initiation of breastfeeding in Ethiopia: A geographically weighted regression analysis
Source: PLoS One. 2022 Sep 15;17(9):e0273793. doi: 10.1371/journal.pone.0273793 (PMC9477376; doi:10.1371/journal.pone.0273793)
Supplement: Attachment — Submitted filename: Point by point respons.docx [file pone.0273793.s003.docx]

**To: PLOS ONE**

From: Samuel Hailegebreal

Subject: **A letter Accompanying Revision in Response to Editors and Reviewer Comments**

**Dear Editors**

The authors would like to thank the editorial team and team of reviewers for constructive and valuable comments. The authors are very happy to submit the second revised version of the manuscript entitled **“Modeling spatial determinants of initiation of breastfeeding in Ethiopia: A geographically weighted regression analysis”** for its publication in your Journal. The comments of the editors and the reviewers were highly insightful and enabled us to greatly improve the quality of our manuscript. In this second revision manuscript we made substantial changes to address your concerns in a point-by-point response. We are very keen to incorporate further comments, if any, for the betterment of the final manuscript.

**Point by Point Response to - Editor Comments**

1. In response to reviewer #2's comment on missing values (see below), you have provided an explanation in the response letter but you have not included this explanation in the "Date management and statistical analysis" section of the Methods. Please could you ensure that you update this section accordingly and then resubmit?

Author response: When a variable has a "missing value," it should have a response but does not, either because the interviewer accidentally left out the question or because the respondent declined to answer. We remove missing values from our analysis by using the STATA drop command in combination with a logical or conditional statement.
